# A new *in situ* method for modifying the Schottky barrier height at buried metal–organic semiconductor interfaces

**DOI:** 10.1093/nsr/nwaf252

**Published:** 2025-06-20

**Authors:** Henning Sirringhaus

**Affiliations:** Cavendish Laboratory, University of Cambridge, UK

**Figure 1.**
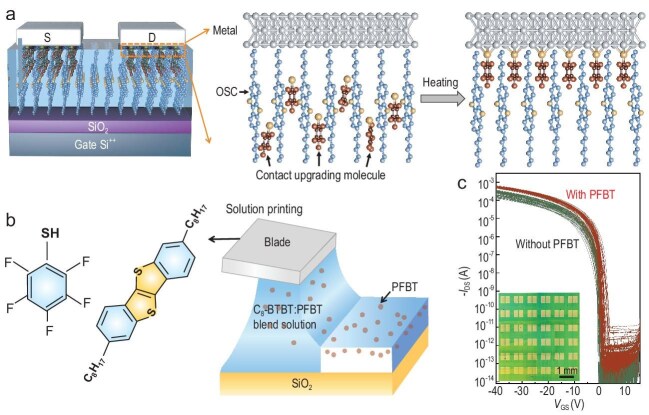
(a) Process for *in situ* modification of the Schottky barrier height at a buried metal–OSC interface. (b) Molecular structure of C_8_-BTBT and PFBT (left) and schematic diagram of the blade-coating process to deposit the SAM/OSC blend solution. (c) Transfer characteristics of an array of 72 OTFTs with and without PFBT (adapted from [Ref. 3]).

Organic thin film transistors (OTFTs) are emerging as a powerful large-area electronic technology for realizing electronic functions on flexible or stretchable substrates for applications in consumer electronics, supply chain management or environmental/biological sensing [1]. In the development of OTFTs, much attention has focused on improving the charge carrier mobilities (*μ*) of the organic semiconductor (OSC). However, an equally important performance metric is the contact resistance at the charge-injecting source-drain contacts: *R_c_*, suitably normalized by the channel width *W*, should be significantly smaller than the normalized channel resistance ${R}_{ch} \cdot W = \ \frac{L}{{\mu {C}_i( {{V}_G - {V}_T} )}}\ $(*C_i_*, capacitance of the gate dielectric; *V_G_*, applied gate voltage; *V_T_*, threshold voltage). In a state-of-the-art OSC, such as the widely studied molecular semiconductor C_8_-BTBT with mobilities of >10 cm^2^/Vs (Fig. 1B), and in devices with typical channel lengths of several micrometers ${R}_{ch} \cdot W$ is on the order of several hundreds of Ωcm, requiring ${R}_c \cdot W$ to be less than 100 Ωcm.

The contact resistance is determined by Schottky barrier formation at the metal–OSC interface and by charge transport in the portion of the OSC that the charges have to cross on their way from the electrodes to the accumulation layer at the gate dielectric interface. A wide range of techniques have been explored to achieve low contact resistance values (for an excellent review see [2]). For p-type OTFTs, a common technique is to minimize the Schottky barrier height by increasing the work function of a gold or silver source-drain electrode to match the often-deep ionization potential of the OSC. This can be achieved by depositing a self-assembled monolayer (SAM), such as perfluorobenzenethiol (PFBT) (Fig. 1b) onto the metal surface prior to OSC deposition. The work function increase is due to the molecular dipole of PFBT pointing away from the thiol group that attaches to the metal surface. A limitation of this approach is that it can only be applied to device configurations, in which the work function modification step is performed before the OSC is deposited on top of the metal electrode, i.e. to bottom-gate, bottom contact as well as top-gate, bottom-contact devices.

Wang *et al.* [3] report an elegant and effective new method to modify the Schottky barrier height at a metal–OSC interface *in situ*, i.e. after the OSC–metal electrode interface is already formed. This makes the technique applicable to all device configurations, including bottom-gate, top-contact configurations (Fig. 1a, left) and may also enable more intimate contact between the modified electrode and the OSC without introducing a van der Waals gap. The method involves (i) mixing the SAM molecule into the solution of the OSC, (ii) forming the metal–OSC interface and (iii) inducing the assembly of the SAM molecule at the metal–OSC interface by a mild annealing step (Fig. 1a, right). Wang *et al.* show that the presence of PFBT during the solution deposition of C_8_-BTBT does not degrade the crystalline microstructure of the thin films, but leads to a significant reduction in the contact resistance to values around 80 Ωcm. This is one of the lowest values reported for C_8_-BTBT, a molecule for which it is difficult to achieve a low contact resistance owing to its deep ionization potential. The reduced contact resistance also benefits other performance metrics; it lowers the subthreshold slope, improves device uniformity and increases the measured field-effect mobility. The technique is also shown to be applicable to devices based on conjugated polymers.

Although some open questions, such as the thermal and operational stability of OTFTs fabricated by this technique, remain to be investigated, the approach demonstrated by Wang *et al.* is likely to become a widely used and powerful method to reduce the contact resistance for a wide range of OSCs in different device architectures.
